# Use of magnetic resonance imaging-guided radiotherapy for breast cancer: a scoping review protocol

**DOI:** 10.1186/s13643-021-01594-9

**Published:** 2021-02-01

**Authors:** Sarah Elliott, Alexandra Berlangieri, Jason Wasiak, Michael Chao, Farshad Foroudi

**Affiliations:** Department of Radiation Oncology, Olivia Newton-John Cancer Wellness and Research Centre, Austin Health, Heidelberg, Victoria Australia

**Keywords:** Breast cancer, MRI-guided radiotherapy, MR simulation, MR linac, Scoping review

## Abstract

**Background:**

In recent years, we have seen the incorporation of magnetic resonance imaging (MRI) simulators into radiotherapy centres and the emergence of the new technology of MR linacs. However, the significant health care resources associated with this advanced technology impact immediate widespread use and availability. There are currently limited studies to demonstrate the clinical effectiveness and inform decision-making on the use of MRI in radiotherapy. The objective of this scoping review is to identify and map the existing evidence surrounding the clinical implementation of MRI-guided radiotherapy in patients with breast cancer*.* It also aims to identify challenges and knowledge gaps in the literature.

**Methods:**

We will perform a comprehensive search in MEDLINE and EMBASE databases from January 2010 onwards. Grey literature sources will include the WHO International Clinical Trials Registry Platform. We will include systematic reviews, randomised and non-randomised controlled studies published in English. Literature should examine the use of magnetic resonance imaging-guided radiotherapy in adults with breast cancer, regardless of cancer stage or severity. Two reviewers will independently screen all titles, abstracts and full-text reports. Data will be extracted and summarised using qualitative (e.g. content and thematic analysis) methods and presented in tables.

**Discussion:**

The results from this review will consolidate the evidence surrounding MRI-guided radiotherapy for breast cancer, contributing to the development and optimisation of patient selection, simulation, planning, treatment delivery, quality assurance and research, to help improve patient outcomes, cancer care and treatment for women with breast cancer.

**Systematic review registration:**

The protocol is available on Open Science Framework at DOI 10.17605/OSF.IO/8TEV6

**Supplementary Information:**

The online version contains supplementary material available at 10.1186/s13643-021-01594-9.

## Background

Breast cancer is the leading cause of cancer death among women. In 2018, it is estimated that there were 647,000 deaths from breast cancer globally, accounting for 15% of all cancer deaths [1]. A majority (approximately 77%) of breast cancer cases are diagnosed as early stage [2].

The standard treatment for early stage breast cancer is breast conservation surgery followed by radiotherapy to the whole breast. Radiotherapy doses of 50 Gy/25 are delivered over 5 weeks and often combined with a boost to the tumour bed to improve local control [3]. The long fractionation time of whole breast irradiation can be a burden to patients, and this has led to hypo-fractionated schemes such as 42.5 Gy/16[4] and 26 Gy/5 [5], which have comparable patient outcomes. An alternative to this is accelerated partial breast irradiation (APBI), which delivers a higher dose of radiotherapy to a limited volume of tissue, with an even shorter course duration. A common regime for external beam and Brachytherapy-based APBI techniques is 38.5 Gy delivered in twice daily fractions over 5 days [6–10]. APBI may be considered for selected cases of lower-risk disease [11, 12] where the tumour bed can be accurately localised.

Contouring breast volumes for radiotherapy is performed on a computed tomography (CT) scan. With limited soft-tissue contrast in CT images, the task is difficult, and high variability in tumour volumes is reported amongst radiation oncologist’s [13–16]. As a result, target volumes may be relatively large, which may lead to higher organ at risk doses and increased risk of acute and late toxicity. For breast radiotherapy treatments, magnetic resonance imaging (MRI) is enabling more sophisticated approaches with the improved accuracy of target definition and the opportunity to evaluate APBI techniques [17–21].

The role of MRI in radiotherapy planning and delivery is increasing [22, 23]. MRI scanners are being introduced as MR simulators. Combination MR Scanners and Linear Accelerators (MR linacs) are a new technology, such as the MRIdian (Viewray, Oakwood, Ohio) and Unity (Elekta, Stockholm, Sweden). They have the ability to provide marker-less, real-time image guidance in radiotherapy treatments, and incorporate functional information through the use of different imaging sequences. Higher precision target localisation combined with dynamic target information can minimise the impact of motion during radiotherapy delivery and lead to decreased planning target volume (PTV) margins and new radiotherapy approaches [24, 25]. Currently, the novel machine has been used on areas including the abdomen, pelvis, head and neck, thorax and oligometastatic disease [26].

Although treatment of the breast may benefit from MRI-guided radiotherapy, implementation for this tumour site is technically challenging and not typically the first choice for clinics new to MRI. Unlike the diagnostic setting, MR simulators are most suitable for target definition. They have physical differences in the magnetic field strength and patient positioning systems, which affect the breast anatomy from how we normally see it. The MR linac is also considerably different to the conventional linac, with mechanical limitations on the collimator, couch, treatment field size and delivery techniques, which change the treatment planning and delivery process for breast radiotherapy. The radiofrequency coils required for MR imaging, associated geometric accuracy and isotropic resolution, and the very presence of a magnetic field during treatment delivery with the effects upon secondary electrons [27–30], are important factors for dose calculations and affect the dosimetry in the breast. Given longer fraction durations on the MR linac, especially with high dose per fraction prescriptions and the adaptive nature of treatments, intrafraction motion must also be considered. Hence, there is a need for some guidance on the implementation of MRI-guided radiotherapy for breast cancer.

### Rationale of the review

MRI-guided radiotherapy is rapidly evolving and expanding to breast treatments [26, 31]. There is the potential to improve the quality of treatments by decreasing toxicity to the breast and adjacent organs, while simplifying the logistics of treatment, which may impact thousands of breast cancer patients annually. The existing literature on this is sparse and scattered. A preliminary search revealed there were no existing scoping review protocols or finalised systematic/scoping reviews on MRI-guided radiotherapy for breast cancer. Therefore, seeking evidence on this topic warrants further exploration. The primary aim of this review is to describe the state of the peer-reviewed literature on key aspects of the radiotherapy chain including simulation, tumour delineation, planning, treatment delivery and quality assurance for MRI-guided breast radiotherapy. A scoping review of available international evidence will help to understand which factors determine the optimal treatment technique, target volume margins, prescription and fractionation schedules, and if APBI is feasible. It will address the main issues related to delivering radiotherapy to the breast site in the presence of a magnetic field. This will draw attention of the radiotherapy community, build momentum and support the development of MRI-guided breast radiotherapy for radiation oncology health services.

### Objectives

In this protocol, we present our methods for conducting a scoping review. The objective of the review is to examine and map the existing literature on the use of the MR simulator and MR linac for radiotherapy of breast cancer among adults, with the aim to identify, summarize and synthesize the existing current knowledge, literature and evidence from published studies. We also aim to identify and analyse potential gaps in the current research base.

## Methods

This protocol has been registered within the Open Science Framework database (registration number: DOI 10.17605/OSF.IO/8TEV6). The proposed scoping review will be reported in accordance with the reporting guidance provided in the Preferred Reporting Items for Systematic Reviews and Meta-Analyses Protocols (PRISMA-P) statement [32, 33] (see checklist in Additional file 1) and the Preferred Reporting Items for Systematic Reviews and Meta-analyses (PRISMA) extension for Scoping Reviews (PRISMA-ScR) [34]. The scoping review methodology will be conducted in accordance with the framework proposed by Arksey and O’Malley [35], which has been further developed by Levac et al. [36]. This framework recommends organising the review process following a series of steps which includes but is not limited to the following: (1) identifying the research question; (2) identifying relevant studies; (3) study selection; (4) charting the data; (5) collating, summarising and reporting the results; and (6) consulting with relevant stakeholders and key informants.

### Stage 1: identifying the research question

This stage consisted of discussion and deliberation with the research team, given the department’s recent acquisition of an MR simulator and MR linac. As mentioned above, the incentive to conduct the review is to scope the existing literature aiming to identify the evidence, map the existing literature and define the optimal use and clinical implementation of the MR simulator and MR linac for breast radiotherapy. More specifically, this research will be guided by the following research question:

*What is the range of existing evidence surrounding the clinical implementation of MRI-guided radiotherapy in patients with breast cancer?*

### Stage 2: identifying relevant studies

#### Eligibility criteria

The Population-Concept-Context (PPC) framework will be used to align the study selection with the research questions. We will restrict our search to English language, peer-reviewed studies. Given the recent emergence of MRI-guided radiotherapy studies, we will also restrict our search to studies published from January 2010 onwards. To be included in the review, sources of evidence will need to be systematic reviews, randomised and non-randomised controlled trials. We will exclude case reports, case series, narrative reviews and animal studies. This review will include studies involving adults (aged ≥ 18 years old) with breast cancer, regardless of stage or severity. They may include pre-operative or post-operative radiotherapy; however, studies on metastatic breast cancer or nodal spread will be specifically excluded. This review will include studies on MRI-guided radiotherapy, such as MR Linac, MR simulation and any type of radiation therapy intervention (e.g. Brachytherapy, VMAT, IMRT, 3DCRT). Studies that report data collected on MR scanners in the diagnostic setting only will be excluded. Studies may have been conducted in any country, but must have taken place in a hospital, clinical or academic setting. Full details of the inclusion and exclusion criteria are summarised in Table 1.

**Table 1:** Study inclusion and exclusion criteria

Study characteristics	Inclusion criteria	Exclusion criteria
Design	Systematic review Randomised and non-randomised controlled studies Clinical Trials	Case report/study Descriptive report
Publication	Peer reviewed journal Published in English Abstract and full text available 2010 to present	Doctoral thesis Conference proceeding, abstract or poster
Participants	Breast cancer only Adults ≥ 18 years Any tumour stage	Any cancer excluding breast metastases, nodal spread
Intervention	MR Linac MR Simulator Any type of radiation therapy intervention, e.g. Brachytherapy, VMAT, IMRT, 3DCRT	Standard diagnostic MRI scans used in a radiation therapy setting

#### Information sources

The primary source of literature will be a structured search of electronic databases MEDLINE (Ovid) and EMBASE (Ovid). Outputs will be included if they are published from 2010 onwards, when MRI-guided radiotherapy studies started to emerge. The secondary source of potentially relevant material will be a search of the ongoing and unidentified clinical trials using the WHO International Clinical Trials Registry Platform search portal. The references of included documents, but also key journals, conference papers, research thesis and dissertations will be hand-searched to identify any additional evidence sources. Efforts will be made to contact authors of completed, ongoing studies, in-press or unpublished literature for information regarding additional studies or relevant material. The search strategy will be designed in conjunction with a research librarian. Key search terms will include breast cancer, MR simulator and MR linac. A draft of the MEDLINE search strategy is outlined in Additional file 2.

### Stage 3: study selection

Two reviewers will independently screen title and abstracts of all potentially relevant citations against the detailed inclusion and exclusion criteria listed in Table 1. The COVIDENCE [37] application will be used for efficient screening. Any disagreement will be resolved by a group discussion. If consensus cannot be achieved, a third review author will be consulted for final study arbitration. We will use the PRISMA flow diagram to outline our search results, depicting the number of studies to be included or excluded from the data analysis phase.

### Stage 4: data charting

Two reviewers will independently extract data from studies fulfilling the inclusion criteria. If any disagreement occurs, this will be resolved by discussion with a third review author. If consensus cannot be achieved, a fourth review author will then be consulted for arbitration. A data extraction form will be developed using Microsoft Excel (Microsoft, Redmond) and reviewed by the review authors before data extraction commences. To ensure data extraction consistency amongst review authors and to ensure the form captures all relevant themes to the scoping review question and objectives, the form will be pilot tested using a number of diverse studies reflecting the nature of the evidence.

The concept of interest of this proposed scoping review is the type, timing, technical and clinical details of MR simulation or MR linac treatments for breast cancer radiotherapy patients. Specifically, we will extract data on the MRI sequences, coils, patient set-up/immobilisation techniques, target margins, tumour localisation, planning and dosimetric issues, adaptive treatments, workflow, safety and quality assurance. Patient outcomes collected will also be of interest. Since a scoping review aims to provide a comprehensive examination of the literature, the data extracted will include, but not be limited to the following information listed in Table 2.

**Table 2:** Planned data extraction headings in the scoping review

Study details	Heading
General study details	Title, lead author, journal, year of publication, type of publication, location, objectives of the article
Study characteristics	Design, duration, number of study arms, measurement methods
Participants	Total number, setting, inclusion/exclusion criteria, age, ethnicity, breast cancer stage
Intervention	Total number of interventions and comparison groups, number of participants in each group, range (variety), stage of radiation therapy process, MR simulator/MR linac
Outcomes	List of outcomes, time-points, data collection method (qualitative/quantitative), scanning parameters, OAR/target delineation, adaptive planning strategies, dose prescription, toxicities, etc.
Results	Results presented by authors, effectiveness, future research directions identified by authors
Challenges	Effect, source, potential solution

### Stage 5: data summary and synthesis of results

Data extracted from the included studies by the review team will be used to develop an analytical framework to collate, summarise and synthesise the data. In particular, we will make use of tables that outline each study according to author, year, setting and implementation stage of the radiotherapy process (i.e. imaging, planning, treatment delivery), as shown in Table 3. These tables will provide an overview of the extent, nature and distribution of the studies included in this review. For example, they will allow comparison of MRI-guided APBI and conventional treatment regimes, as well as MRI magnet strength and doses to organs at risk (OAR). Furthermore, themes identified from all the studies will be organised, coded and thematically analysed to showcase areas of clinical practice using a narrative summary format.

**Table 3:** Proposed categories for presentation

Table heading	Key results
Implementation 1: imaging	Patient selection MR acquisition sequences Image quality Quality assurance developments Image registration Patient setup Immobilisation equipment
Implementation 2: planning	Structure delineation Technique Beam properties Dose calculation Dosimetry Corrections Inter-fractional and intra-fractional adaptions Target volume margins Dose coverage of target Organ at risk doses Skin dose
Implementation 3: treatment	Motion management Tumour localisation Delivered vs planned dose Workflow quality assurance and delivery issues Safety Patient outcomes Early assessment of treatment response
Challenges/knowledge gaps	Commissioning Planning Delivery Future research Clinical trials enabled by MRI-guided radiotherapy for breast cancer Limitations of technological development

### Stage 6: consultation

We will use this opportunity to share our findings and refer to relevant health care professionals including radiation therapists, physicists and radiation oncologists to gain more insight into our data from different perspectives. Information gleaned from our clinical stakeholders will be used to allow for any adjustments or expansion to the initial analytical framework.

## Discussion

The study will contain information gathered from already published papers; therefore, it does not require ethical approval.

We aim to conduct a well-structured and reproducible study through rigorous planning and thorough documentation of the study methodology, creating an optimal framework for data charting, appraisal and synthesis. In performing this study, we anticipate one operational challenge. As part of the inclusion criteria we will include studies with a seemingly wide range of methodology and research objectives. While this will not affect the title and abstract review or full-text screening process, data synthesis and collation of a wide range of evidence in a harmonised way will be difficult.

MRI-guided radiotherapy is an advanced technology that requires significant health care resources including cost, a large physical space and clinical expertise to perform the diverse and complex tasks associated with MRI. As such, the emerging technology is not available to everyone. There are a limited number of studies to inform decision-making in the use of MRI-guided therapies for breast cancer and to demonstrate clinical effectiveness to this treatment site. Evaluating the gathered studies for this new technology poses a particular challenge in the limited number of published studies and participants to date. We may not be able to make definitive conclusions if sample sizes for any specific topic are too small, as this will decrease the power of the study.

Although it is not a limitation, we will not assess quality of the evidence during the review process.

Any amendments made to this protocol when conducting the study will be outlined and reported in outputs of the review results and the final manuscript. Following completion of data extraction, the dissemination process will start with sharing the initial findings of this review with other researchers, radiation oncologists, physicists, radiation therapists and MR radiographers within and beyond our organisation to enhance the review’s quality. The findings will serve as a tool for implementing MRI-guided radiotherapy in Australia. We will submit study findings to a peer-reviewed scientific journal. In addition, we will seek to present the study at scientific conferences.

Consolidation of the evidence surrounding MRI-guided radiotherapy for breast cancer from this scoping review will contribute to the knowledge base of health professionals. This knowledge can support the development and optimisation of patient selection, simulation, planning, treatment delivery, quality assurance and research. Furthermore, it can accelerate the implementation of new approaches to breast radiotherapy, which could help improve patient outcomes, cancer care and treatment for women with breast cancer.

## Supplementary Information

**Additional file 1:.** PRISMA-ScR checklist. Completed checklist of recommended items to include in a systematic review.

**Additional file 2:.** MEDLINE search strategy. The example search strategy to be used in the MEDLINE database.

## Data Availability

Not applicable

## Abbreviations

MRI: Magnetic resonance imaging
MR: Magnetic resonance
APBI: Accelerated partial breast irradiation
CT: Computed tomography
PRISMA-ScR: Preferred Reporting Items for Systematic reviews and Meta-Analysis extension for Scoping Reviews
VMAT: Volume modulated arc therapy
IMRT: Intensity modulated radiation therapy
3DCRT: Three dimensional conformal radiation therapy
OAR: Organs at risk
