# Supplementary material for: Use of magnetic resonance imaging-guided radiotherapy for breast cancer: a scoping review protocol
Source: Syst Rev. 2021 Feb 1;10:44. doi: 10.1186/s13643-021-01594-9 (PMC7852080; doi:10.1186/s13643-021-01594-9)
Supplement: Supplementary file 2 — Additional file 2:. MEDLINE search strategy. The example search strategy to be used in the MEDLINE database. [file 13643_2021_1594_MOESM2_ESM.docx]

**Additional file 2: MEDLINE search example**

Database: Ovid MEDLINE(R) ALL 1946 to January 08, 2021

Search Strategy:

| **#** | **Searches** |
| --- | --- |
| 1 | exp Radiotherapy, Adjuvant/ |
| 2 | exp Radiotherapy/ |
| 3 | exp Radiation Oncology/ |
| 4 | (radiother$ or radiat$ or irradiat$ or radiochemo$ or chemoradiat$).mp. |
| 5 | (external beam and (radiation or radiotherapy or irradiation)).tw. |
| 6 | (three dimensional and (radiation or radiotherapy or irradiation)).tw. |
| 7 | (3D and (radiation or radiotherapy or irradiation)).tw. |
| 8 | (3D conformal and (radiation or radiotherapy or irradiation)).tw. |
| 9 | (three dimensional conformal and (radiation or radiotherapy or irradiation)).tw. |
| 10 | (whole breast and (radiation or radiotherapy or irradiation)).tw. |
| 11 | exp Radiotherapy, Conformal/ |
| 12 | Radiotherapy, Intensity-Modulated/ |
| 13 | exp brachytherapy/ |
| 14 | Radiotherapy, Computer-Assisted/ |
| 15 | exp radiotherapy, high energy/ |
| 16 | exp radiotherapy, image guided/ |
| 17 | (interstitial and (radiation or radiotherapy or irradiation)).tw. |
| 18 | (electron and (radiation or radiotherapy or irradiation)).tw. |
| 19 | (photon and (radiation or radiotherapy or irradiation)).tw. |
| 20 | or/1-18 |
| 21 | exp Breast Neoplasms/ |
| 22 | (breast adj6 cancer$).tw. |
| 23 | (breast adj6 neoplasm$).tw. |
| 24 | (breast adj6 carcinoma$).tw. |
| 25 | (breast adj6 tumo?r$).tw. |
| 26 | or/21-25 |
| 27 | exp Magnetic Resonance Imaging/ |
| 28 | ((Magnetic and resonance) or MR*).mp. |
| 29 | MRI Linac.tw. |
| 30 | Particle Accelerators/ |
| 31 | Linear Accelerators.mp. |
| 32 | or/27-31 |
| 33 | 20 and 26 and 32 |
| 34 | limit 33 to (english language and humans) |
| 35 | limit 34 to yr="2010 -Current" |
| 36 | limit 35 to dt=20190621-20210211 [21 June 2021 to 11 January 21] |
